# Crystal structures of the isomeric dipeptides l-glycyl-l-me­thio­nine and l-me­thionyl-l-glycine

**DOI:** 10.1107/S2056989024005504

**Published:** 2024-06-14

**Authors:** Sainath Babu, Frank R. Fronczek, Rao M. Uppu, Michelle O. Claville

**Affiliations:** a Department of Biological Sciences, School of Science, Hampton University, Hampton, VA, 23669, USA; b Department of Chemistry Louisiana State University,Baton Rouge LA 70803 USA; c Department of Environmental Toxicology Southern University and A&M College Baton Rouge LA 70813 USA; d School of Science, Hampton University, Hampton, VA, 23669, USA; University of Aberdeen, United Kingdom

**Keywords:** crystal structure, dipeptide, me­thionyl oxidation, absolute configuration

## Abstract

The oxidation of me­thionyl peptides can contribute to increased biological (oxidative) stress and development of various inflammatory diseases. The conformation of peptides has an important role in the mechanism of oxidation and the inter­mediates formed in the reaction. Herein, the crystal structures of the isomeric dipeptides Gly-Met (Gly = glycine and Met = me­thio­nine) and Met-Gly, both C_7_H_14_N_2_O_3_S, are reported. Both mol­ecules exist in the solid state as zwitterions with nominal proton transfer from the carb­oxy­lic acid to the primary amine group. The Gly-Met mol­ecule has an extended backbone structure, while Met-Gly has two nearly planar regions kinked at the C atom bearing the NH_3_ group. In the crystals, both structures form extensive three-dimensional hydrogen-bonding networks *via* N—H⋯O and bifurcated N—H⋯(O,O) hydrogen bonds having N⋯O distances in the range 2.6619 (13)–2.8513 (13) Å for Gly-Met and 2.6273 (8)–3.1465 (8) Å for Met-Gly.

## Abstract 2

The zwitterionic, isomeric, title compounds, Gly-Met and Met-Gly are me­thio­nine-containing dipeptides, which show very different conformations in the solid state.

## 1. Chemical context

Me­thio­nine and me­thionyl peptides play an important role in protein oxidation. The sulfur atom in me­thio­nine can easily be oxidized by free radicals or oxidants and lead to sulfur radical cations (Bobrowski & Holcman, 1989 ▸) or a corresponding sulfoxide (Schöneich, 2005 ▸). The formation of reactive radical cations is responsible for me­thionyl protein damage and misconformation, which has been implicated in numerous inflammatory (Vogt, 1995 ▸) and age-related diseases (Schöneich, 2005 ▸; Stadtman *et al.*, 2005 ▸).
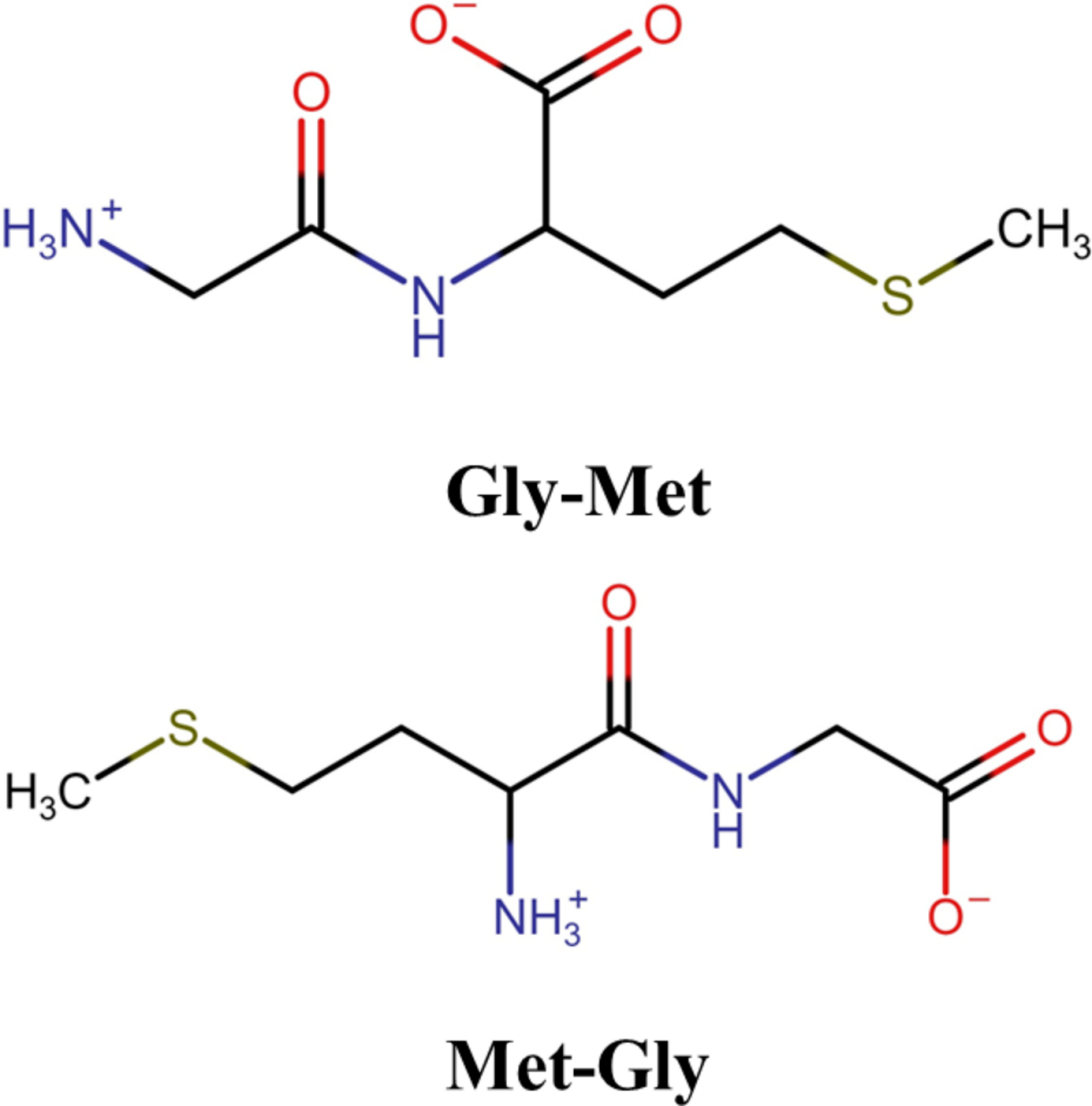


Gly-Met (Gly = glycine, Met = me­thio­nine) and its reverse sequence, Met-Gly, are two simple dipeptides. It has been shown that the position of me­thio­nine with respect to the N-terminus of the peptide determines the mechanism of oxidation of me­thionyl peptides. For instance, in photosensitized oxidation reactions, substantial amounts of radical cations stabilized with sulfur–nitro­gen (S∴N) three-electron bonded species were observed with Met-Gly, while similar stabilization of the radical cations was not observed with Gly-Met (Pedzinski *et al.*, 2009 ▸). In collision-induced radical dissociation, Gly-Met leads to the loss of a CH_3_—S—CH=CH_2_ fragment from the peptide, while such dissociation in Met-Gly leads to the loss of a CH_3_S^**·**^ (Lau *et al.*, 2013 ▸) radical. Thus, the difference in me­thio­nine position leads to quite different reaction inter­mediates, which may eventually affect the stability of the peptide and its biological function.

The oxidation of me­thio­nine peptides is determined by several factors, including peptide structure, the position of me­thio­nine within the sequence, neighboring groups to me­thio­nine, nature of the oxidants, and solvent properties. The conformation of the peptide is also an important factor that needs to be considered to understand the mechanism of oxidation. Peptides can exist in either cationic, zwitterionic, or anionic conformations, depending on the solvent and the pH. The present report describes the zwitterionic structures of Gly-Met and Met-Gly dipeptides. We believe that some of the differences observed in the literature related to me­thionyl peptide oxidations could be attributed to the conformation of the peptide in solution.

## 2. Structural commentary

**Figure 1:**
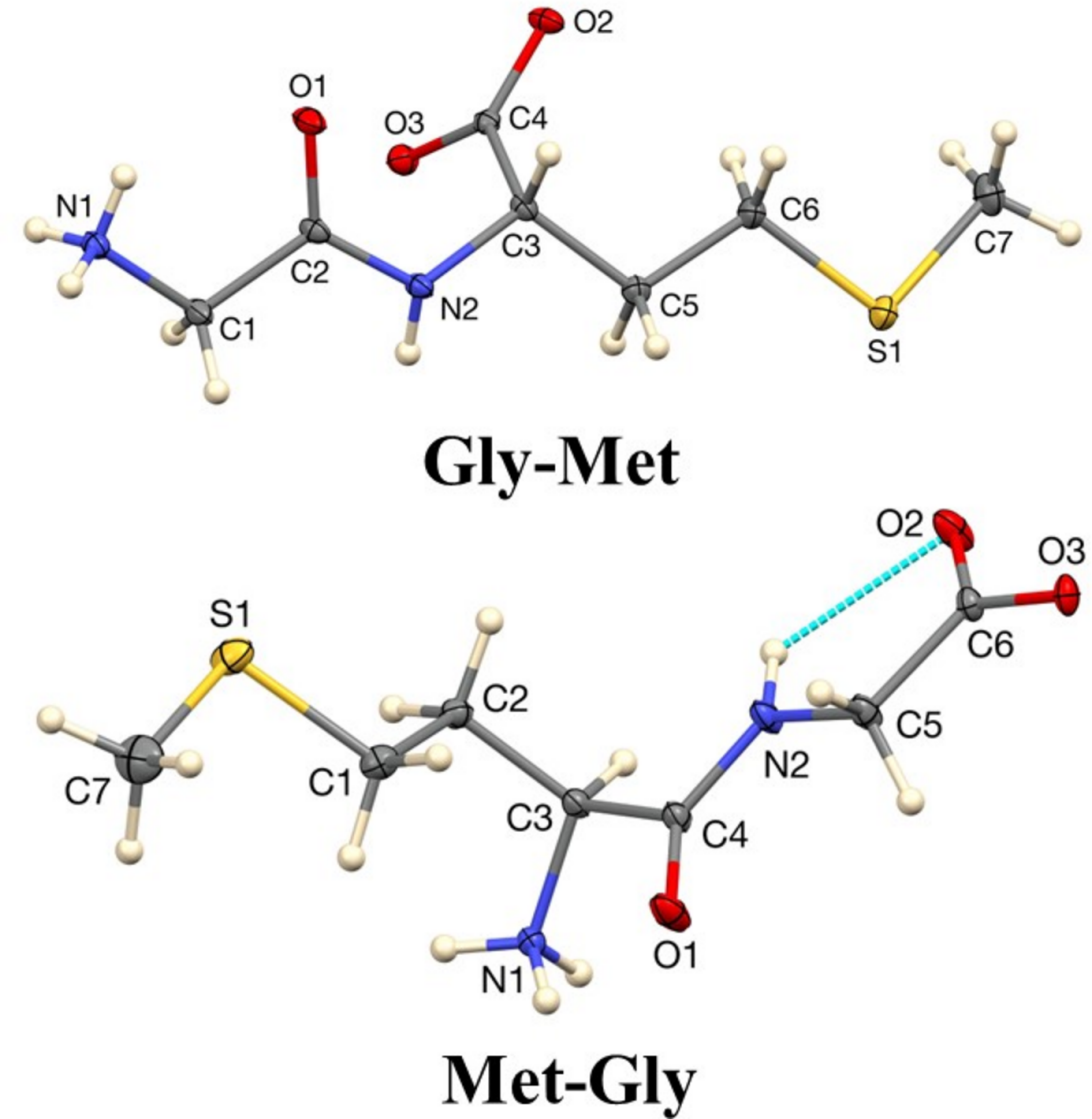
The mol­ecular structures of Gly-Met and Met-Gly showing 50% displacement ellipsoids.

Both of the dipeptides are in their zwitterionic forms in the solid state (Fig. 1 ▸). The amide N1 atom of both Gly-Met and Met-Gly is in the protonated NH_3_^+^ form, and the C6/O2/O3 carb­oxy­lic groups are in their deprotonated (COO)^−^ forms, as evidenced by the C—O distances of 1.2598 (13) and 1.2546 (13) Å in Gly-Met and 1.2534 (9) and 1.2635 (8) Å in Met-Gly. The C—NH_3_ distance is 1.4809 (14) Å in Gly-Met and 1.4855 (8) in Met-Gly. The backbone of the Gly-Met mol­ecule is extended, with its six torsion angle magnitudes in the range 163.44 (9)–177.94 (8)°. Thus, the ten atoms of the chain are close to coplanar with a mean deviation of 0.091 Å. The backbone of the Met-Gly mol­ecule is substanti­ally kinked at C3. The eight-atom segment containing the carboxyl­ate group is planar to within a mean deviation of 0.056 Å, and the five-atom segment containing the S atom is planar to within a mean deviation of 0.086 Å. These two planes inter­sect at C3, forming a dihedral angle of 70.502 (13)°. The absolute configurations of both mol­ecules were confirmed from their refined Flack parameters (Parsons *et al.*, 2013 ▸), with values of 0.02 (2) for Gly-Met and 0.011 (11) for Met-Gly.

## 3. Supra­molecular features

**Figure 2:**
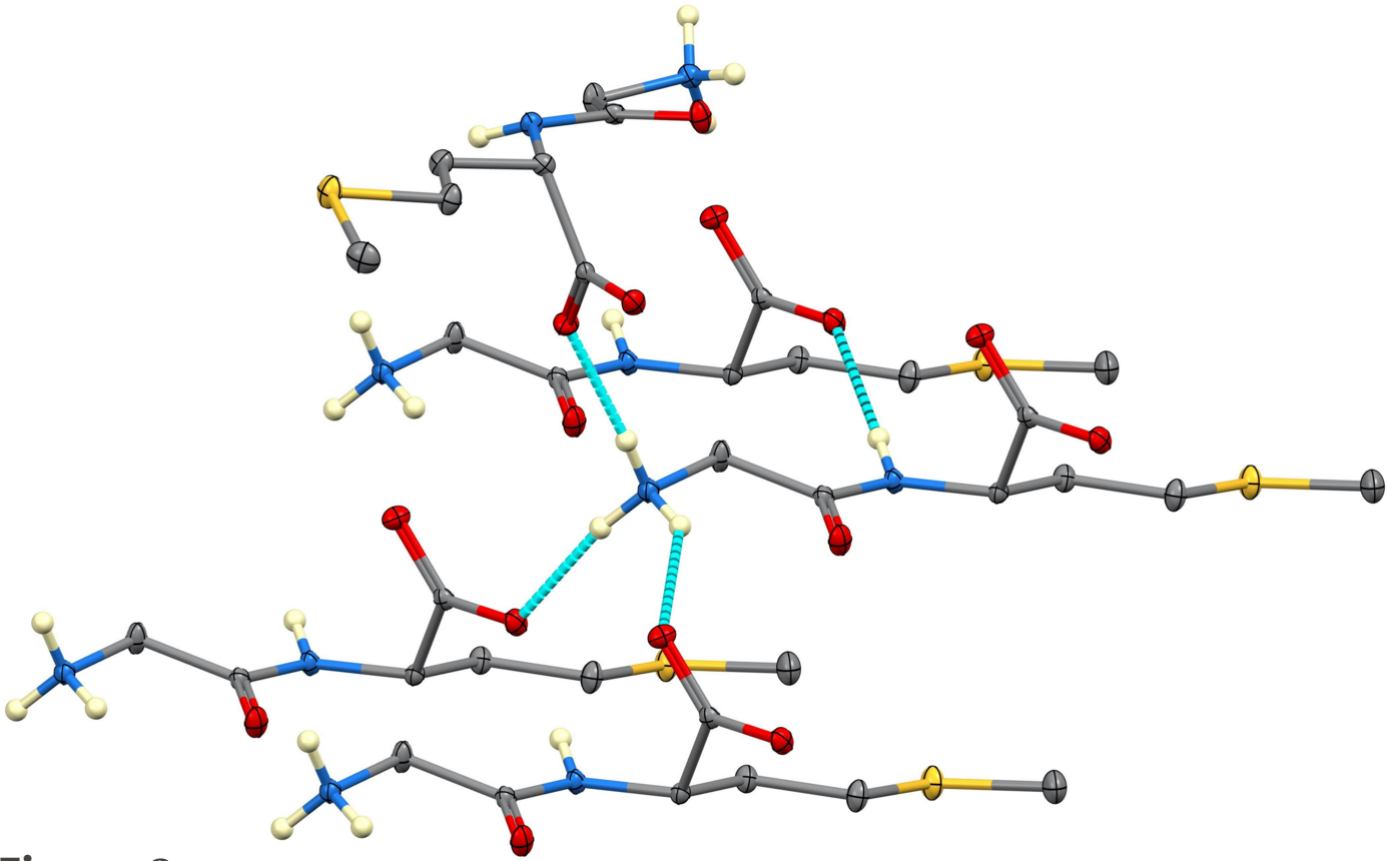
Hydrogen bonding in Gly-Met. H atoms on C are not shown.

**Figure 3:**
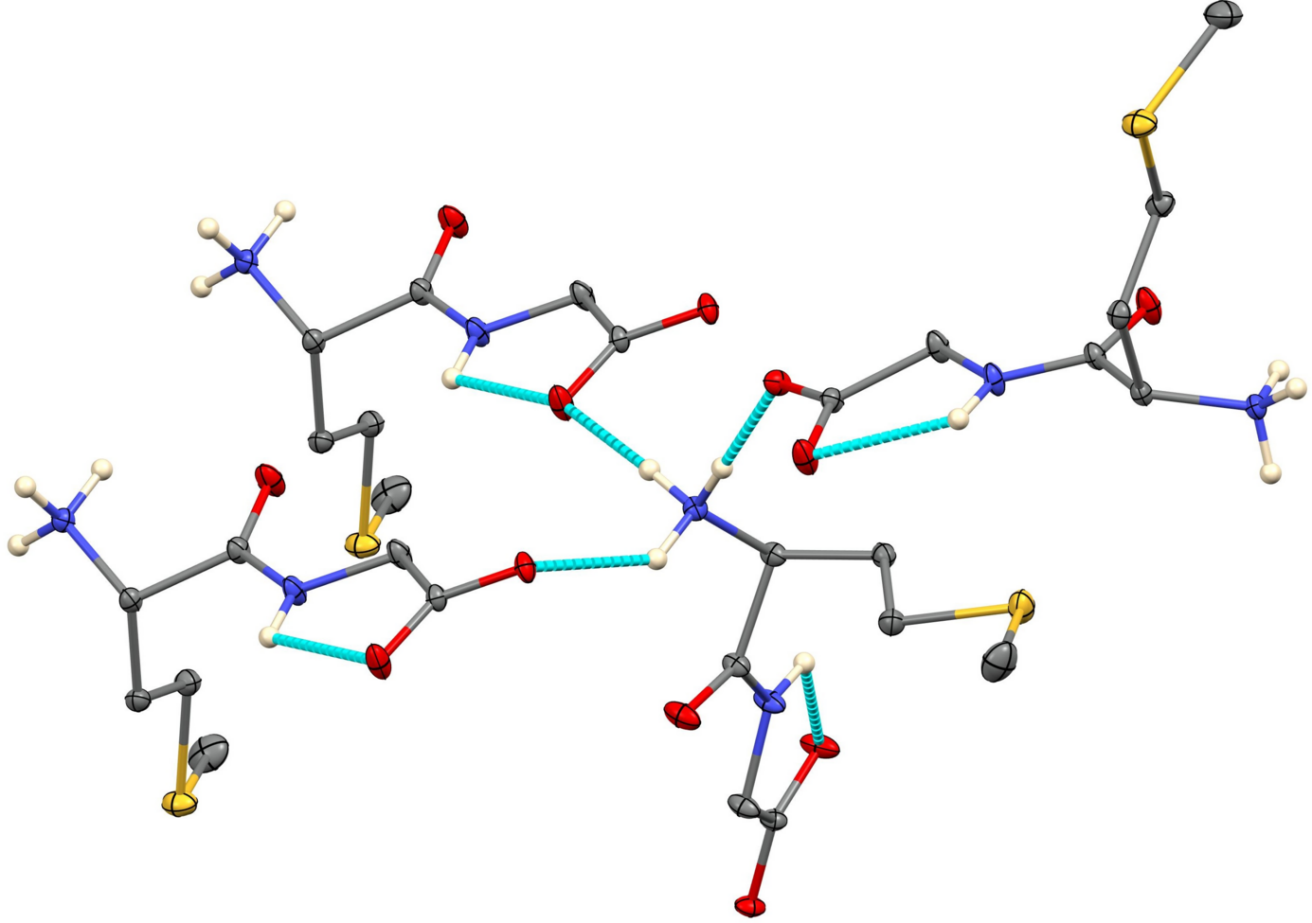
Hydrogen bonding in Met-Gly. H atoms on C are not shown.

Inter­molecular inter­actions in both structures are dominated by N—H⋯O hydrogen bonds, some of which are bifurcated. In Gly-Met (Table 1 ▸), the NH_3_^+^ group donates hydrogen bonds to three separate mol­ecules, and the N—H group donates to a fourth (Fig. 2 ▸), forming a complex three-dimensional array of hydrogen bonds. Graph sets (Etter *et al.*, 1990 ▸) include 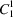
(5) and 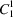
(8) chains and 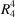
(22) loops. In Met-Gly (Table 2 ▸), the NH_3_^+^ group also donates hydrogen bonds to three different mol­ecules (Fig. 3 ▸), but the N—H group does not participate in inter­molecular inter­actions and makes a very non-linear [N—H⋯O = 110.6 (10)°] intra­molecular contact to an O atom of the carboxyl­ate group. Nevertheless, the hydrogen-bonding array is three-dimensional, and graph sets include 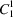
(8) chains, 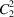
(6) chains and 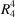
(22) loops.

## 4. Database survey

A search of the Cambridge Structural Database (CSD, version 5.45, Update 1, March 2024; Groom *et al.*, 2016 ▸) revealed that no crystal structures were reported for either Gly-Met or Met-Gly. Other dipeptides containing me­thio­nine have been reported, including l-Ala-l-Met hemihydrate (Gorbitz, 2003 ▸), dl-Ala-ld-Met (Jha *et al.*, 2020 ▸), l-Pro-l-Met monohydrate (Padmanabhan & Yadava, 1983 ▸), l-Met-l-Met (Stenkamp & Jensen, 1975 ▸), l-Met-l-Ala (Gorbitz, 2000 ▸), and l-Met-l-Ser (Gorbitz *et al.*, 2006 ▸).

## 5. Synthesis and crystallization

The dipeptides Gly-Met and Met-Gly were obtained commercially (Chem-impex Inter­national Inc., Wood Dale, IL, USA). A supersaturated solution of each dipeptide was prepared in a small test tube by mixing the compound with warm methanol. Approximately 100 mg of dipeptide was added to 10 ml of methanol and additional solvent was added slowly in small increments with agitation and keeping the test tube at 333 K in a water bath. The solutions were allowed to cool and left undisturbed at room temperature over two weeks for slow evaporation and crystallization to yield colorless plates of Gly-Met and large colorless needles of Met-Gly.

## 6. Refinement

Crystal data, data collection and structure refinement details are summarized in Table 3 ▸. All H atoms were located in difference maps and those on carbon were treated as riding in geometrically idealized positions with C—H distances = 1.00 Å for *R*_3_CH, 0.99 Å for CH_2_ and 0.98 Å for methyl. *U*_iso_(H) values were assigned as 1.2*U*_eq_ for the attached C atom (1.5*U*_eq_ for meth­yl). The positions of the H atoms attached to N atoms were refined. Their *U*_iso_ values were assigned as 1.2*U*_eq_ for the NH groups and 1.5*U*_eq_ for NH_3_.

## Supplementary Material

Crystal structure: contains datablock(s) Gly-Met, Met-Gly, global. DOI: 10.1107/S2056989024005504/hb8099sup1.cif

Structure factors: contains datablock(s) Gly-Met. DOI: 10.1107/S2056989024005504/hb8099Gly-Metsup2.hkl

Structure factors: contains datablock(s) Met-Gly. DOI: 10.1107/S2056989024005504/hb8099Met-Glysup3.hkl

CCDC references: 2361459, 2361458

Additional supporting information:  crystallographic information; 3D view; checkCIF report

## Figures and Tables

**Table 1:** Hydrogen-bond geometry (Å, °) for Gly-Met[Chem scheme1]

*D*—H⋯*A*	*D*—H	H⋯*A*	*D*⋯*A*	*D*—H⋯*A*
N1—H11*N*⋯O3^i^	0.83 (2)	2.15 (2)	2.8513 (13)	143.0 (18)
N1—H11*N*⋯O1	0.83 (2)	2.15 (2)	2.6619 (13)	120.1 (16)
N1—H12*N*⋯O2^ii^	0.886 (19)	1.850 (19)	2.7275 (12)	170.7 (18)
N1—H13*N*⋯O3^iii^	0.849 (19)	1.86 (2)	2.7006 (13)	168 (2)
N2—H2*N*⋯O2^iv^	0.832 (19)	2.000 (19)	2.8225 (12)	170.0 (18)

Symmetry codes: (i) 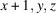; (ii) 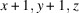; (iii) 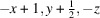; (iv) 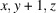.

**Table 2:** Hydrogen-bond geometry (Å, °) for Met-Gly[Chem scheme1]

*D*—H⋯*A*	*D*—H	H⋯*A*	*D*⋯*A*	*D*—H⋯*A*
N1—H11*N*⋯O2^i^	0.885 (14)	1.854 (14)	2.7290 (8)	169.5 (13)
N1—H11*N*⋯O3^i^	0.885 (14)	2.593 (15)	3.1465 (8)	121.4 (11)
N1—H12*N*⋯O3^ii^	0.896 (13)	1.857 (13)	2.7531 (7)	179.6 (14)
N1—H13*N*⋯O3^iii^	0.907 (14)	2.015 (14)	2.7529 (8)	137.5 (11)
N1—H13*N*⋯O1	0.907 (14)	2.211 (13)	2.6940 (8)	112.7 (10)
N2—H2*N*⋯O2	0.879 (15)	2.185 (13)	2.6273 (8)	110.6 (10)

Symmetry codes: (i) 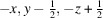; (ii) 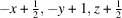; (iii) 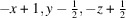.

**Table 3:** Experimental details

	Gly-Met	Met-Gly
Crystal data		
Chemical formula	C_7_H_14_N_2_O_3_S	C_7_H_14_N_2_O_3_S
*M*_r_	206.26	206.26
Crystal system, space group	Monoclinic, *P*2_1_	Orthorhombic, *P*2_1_2_1_2_1_
Temperature (K)	90	90
*a*, *b*, *c* (Å)	6.2517 (2), 5.4935 (2), 14.5686 (6)	5.2521 (3), 11.4126 (7), 16.5403 (10)
α, β, γ (°)	90, 91.147 (4), 90	90, 90, 90
*V* (Å^3^)	500.24 (3)	991.43 (10)
*Z*	2	4
Radiation type	Mo *K*α	Mo *K*α
μ (mm^−1^)	0.30	0.31
Crystal size (mm)	0.38 × 0.29 × 0.03	0.25 × 0.24 × 0.17

Data collection		
Diffractometer	Bruker Kappa APEXII DUO CCD	Bruker Kappa APEXII DUO CCD
Absorption correction	Multi-scan (*SADABS*; Krause *et al.*, 2015 ▸)	Multi-scan (*SADABS*; Krause *et al.*, 2015 ▸)
*T*_min_, *T*_max_	0.836, 0.991	0.872, 0.950
No. of measured, independent and observed [*I* > 2σ(*I*)] reflections	11393, 5922, 5356	33593, 6231, 5939
*R*_int_	0.026	0.030
(sin θ/λ)_max_ (Å^−1^)	0.909	0.909

Refinement		
*R*[*F*^2^ > 2σ(*F*^2^)], *wR*(*F*^2^), *S*	0.033, 0.079, 1.04	0.023, 0.061, 1.06
No. of reflections	5922	6231
No. of parameters	131	131
No. of restraints	1	0
H-atom treatment	H atoms treated by a mixture of independent and constrained refinement	H atoms treated by a mixture of independent and constrained refinement
Δρ_max_, Δρ_min_ (e Å^−3^)	0.53, −0.30	0.44, −0.20
Absolute structure	Flack *x* determined using 2141 quotients [(*I*^+^)−(*I*^−^)]/[(*I*^+^)+(*I*^−^)] Parsons *et al.* (2013 ▸)	Flack *x* determined using 2494 quotients [(*I*^+^)−(*I*^−^)]/[(*I*^+^)+(*I*^−^)] Parsons *et al.* (2013 ▸)
Absolute structure parameter	0.02 (2)	0.011 (11)

Computer programs: *APEX2* and *SAINT* (Bruker, 2016 ▸), *SHELXS97* (Sheldrick, 2008 ▸), *SHELXL2018/1* (Sheldrick, 2015 ▸), *Mercury* (Macrae *et al.*, 2020 ▸), and *publCIF* (Westrip, 2010 ▸).

## supplementary crystallographic information

### L-Glycyl-L-methionine (Gly-Met) Crystal data

**Table:**

C_7_H_14_N_2_O_3_S	*F*(000) = 220
*M**_r_* = 206.26	*D*_x_ = 1.369 Mg m^−^^3^
Monoclinic, *P*2_1_	Mo *K*α radiation, λ = 0.71073 Å
*a* = 6.2517 (2) Å	Cell parameters from 5067 reflections
*b* = 5.4935 (2) Å	θ = 2.8–40.1°
*c* = 14.5686 (6) Å	µ = 0.30 mm^−^^1^
β = 91.147 (4)°	*T* = 90 K
*V* = 500.24 (3) Å^3^	Plate, colourless
*Z* = 2	0.38 × 0.29 × 0.03 mm

### L-Glycyl-L-methionine (Gly-Met) Data collection

**Table:**

Bruker Kappa APEXII DUO CCD diffractometer	5922 independent reflections
Radiation source: fine-focus sealed tube	5356 reflections with *I* > 2σ(*I*)
TRIUMPH curved graphite monochromator	*R*_int_ = 0.026
φ and ω scans	θ_max_ = 40.3°, θ_min_ = 2.8°
Absorption correction: multi-scan (SADABS; Krause *et al.*, 2015)	*h* = −9→11
*T*_min_ = 0.836, *T*_max_ = 0.991	*k* = −9→9
11393 measured reflections	*l* = −26→26

### L-Glycyl-L-methionine (Gly-Met) Refinement

**Table:**

Refinement on *F*^2^	Hydrogen site location: mixed
Least-squares matrix: full	H atoms treated by a mixture of independent and constrained refinement
*R*[*F*^2^ > 2σ(*F*^2^)] = 0.033	*w* = 1/[σ^2^(*F*_o_^2^) + (0.0417*P*)^2^] where *P* = (*F*_o_^2^ + 2*F*_c_^2^)/3
*wR*(*F*^2^) = 0.079	(Δ/σ)_max_ = 0.001
*S* = 1.04	Δρ_max_ = 0.53 e Å^−^^3^
5922 reflections	Δρ_min_ = −0.30 e Å^−^^3^
131 parameters	Absolute structure: Flack *x* determined using 2141 quotients [(*I*^+^)-(*I*^-^)]/[(*I*^+^)+(*I*^-^)] Parsons *et al.* (2013)
1 restraint	Absolute structure parameter: 0.02 (2)

### L-Glycyl-L-methionine (Gly-Met) Special details

**Table:**

Geometry. All esds (except the esd in the dihedral angle between two l.s. planes) are estimated using the full covariance matrix. The cell esds are taken into account individually in the estimation of esds in distances, angles and torsion angles; correlations between esds in cell parameters are only used when they are defined by crystal symmetry. An approximate (isotropic) treatment of cell esds is used for estimating esds involving l.s. planes.

### L-Glycyl-L-methionine (Gly-Met) Fractional atomic coordinates and isotropic or equivalent isotropic displacement parameters (Å^2^)

**Table:**

	*x*	*y*	*z*	*U*_iso_*/*U*_eq_	
S1	−0.20146 (4)	0.44474 (6)	0.42848 (2)	0.01468 (6)	
O1	0.69310 (13)	0.51962 (16)	0.16278 (6)	0.01361 (15)	
O2	0.19251 (13)	0.15688 (15)	0.17287 (6)	0.01189 (14)	
O3	0.17853 (13)	0.50896 (16)	0.09597 (6)	0.01188 (13)	
N1	0.87880 (15)	0.89936 (16)	0.08355 (7)	0.00999 (15)	
H11N	0.913 (3)	0.757 (4)	0.0948 (13)	0.015*	
H12N	0.981 (3)	0.994 (4)	0.1072 (12)	0.015*	
H13N	0.865 (3)	0.912 (4)	0.0257 (13)	0.015*	
N2	0.42247 (14)	0.73584 (17)	0.22578 (7)	0.00962 (14)	
H2N	0.353 (3)	0.863 (4)	0.2171 (13)	0.012*	
C1	0.67091 (16)	0.9440 (2)	0.12752 (8)	0.01317 (16)	
H1D	0.563279	0.995080	0.080609	0.016*	
H1E	0.686342	1.076254	0.173329	0.016*	
C2	0.59716 (16)	0.71258 (19)	0.17425 (7)	0.00938 (15)	
C3	0.30874 (16)	0.51654 (19)	0.25172 (7)	0.00890 (15)	
H3	0.410296	0.406010	0.285399	0.011*	
C4	0.22080 (16)	0.38311 (18)	0.16587 (7)	0.00835 (15)	
C5	0.12523 (18)	0.5846 (2)	0.31603 (8)	0.01112 (17)	
H5A	0.184307	0.675550	0.369448	0.013*	
H5B	0.023082	0.692558	0.282903	0.013*	
C6	0.00678 (19)	0.3600 (2)	0.34991 (8)	0.01315 (18)	
H6A	0.109333	0.249024	0.381309	0.016*	
H6B	−0.057507	0.272165	0.296822	0.016*	
C7	−0.3286 (2)	0.1514 (2)	0.43812 (9)	0.0175 (2)	
H7A	−0.381335	0.098919	0.377488	0.026*	
H7B	−0.448752	0.163281	0.480064	0.026*	
H7C	−0.224743	0.032488	0.462053	0.026*	

### L-Glycyl-L-methionine (Gly-Met) Atomic displacement parameters (Å^2^)

**Table:**

	*U*^11^	*U*^22^	*U*^33^	*U*^12^	*U*^13^	*U*^23^
S1	0.01416 (11)	0.01407 (11)	0.01605 (12)	−0.00108 (10)	0.00637 (8)	−0.00091 (10)
O1	0.0117 (3)	0.0077 (3)	0.0216 (4)	0.0011 (3)	0.0036 (3)	0.0016 (3)
O2	0.0114 (3)	0.0072 (3)	0.0170 (4)	−0.0006 (2)	−0.0009 (3)	−0.0006 (3)
O3	0.0144 (3)	0.0112 (3)	0.0100 (3)	0.0016 (3)	−0.0009 (2)	0.0005 (3)
N1	0.0094 (3)	0.0088 (4)	0.0118 (4)	−0.0009 (3)	0.0007 (3)	0.0011 (3)
N2	0.0087 (3)	0.0068 (3)	0.0133 (4)	−0.0008 (3)	0.0007 (3)	0.0006 (3)
C1	0.0119 (4)	0.0079 (3)	0.0199 (4)	0.0005 (4)	0.0050 (3)	0.0024 (4)
C2	0.0085 (4)	0.0074 (3)	0.0121 (4)	−0.0012 (3)	−0.0009 (3)	0.0003 (3)
C3	0.0086 (3)	0.0077 (3)	0.0104 (4)	−0.0009 (3)	0.0001 (3)	0.0014 (3)
C4	0.0068 (3)	0.0076 (3)	0.0107 (4)	0.0006 (3)	0.0011 (3)	−0.0006 (3)
C5	0.0121 (4)	0.0104 (4)	0.0110 (4)	−0.0013 (3)	0.0021 (3)	−0.0007 (3)
C6	0.0141 (4)	0.0113 (4)	0.0142 (4)	−0.0011 (3)	0.0054 (3)	−0.0003 (3)
C7	0.0147 (5)	0.0183 (5)	0.0194 (5)	−0.0046 (4)	0.0030 (4)	0.0025 (4)

### L-Glycyl-L-methionine (Gly-Met) Geometric parameters (Å, º)

**Table:**

S1—C7	1.8037 (13)	C1—H1D	0.9900
S1—C6	1.8114 (11)	C1—H1E	0.9900
O1—C2	1.2310 (13)	C3—C4	1.5415 (15)
O2—C4	1.2598 (13)	C3—C5	1.5417 (15)
O3—C4	1.2546 (13)	C3—H3	1.0000
N1—C1	1.4809 (14)	C5—C6	1.5262 (16)
N1—H11N	0.83 (2)	C5—H5A	0.9900
N1—H12N	0.886 (19)	C5—H5B	0.9900
N1—H13N	0.849 (19)	C6—H6A	0.9900
N2—C2	1.3437 (14)	C6—H6B	0.9900
N2—C3	1.4527 (14)	C7—H7A	0.9800
N2—H2N	0.832 (19)	C7—H7B	0.9800
C1—C2	1.5181 (16)	C7—H7C	0.9800
			
C7—S1—C6	98.22 (6)	C4—C3—H3	108.8
C1—N1—H11N	107.2 (13)	C5—C3—H3	108.8
C1—N1—H12N	111.5 (12)	O3—C4—O2	125.57 (10)
H11N—N1—H12N	107.1 (19)	O3—C4—C3	117.55 (9)
C1—N1—H13N	110.1 (12)	O2—C4—C3	116.86 (9)
H11N—N1—H13N	107.3 (19)	C6—C5—C3	111.85 (9)
H12N—N1—H13N	113.3 (18)	C6—C5—H5A	109.2
C2—N2—C3	118.29 (9)	C3—C5—H5A	109.2
C2—N2—H2N	115.1 (13)	C6—C5—H5B	109.2
C3—N2—H2N	118.5 (13)	C3—C5—H5B	109.2
N1—C1—C2	109.41 (9)	H5A—C5—H5B	107.9
N1—C1—H1D	109.8	C5—C6—S1	110.86 (8)
C2—C1—H1D	109.8	C5—C6—H6A	109.5
N1—C1—H1E	109.8	S1—C6—H6A	109.5
C2—C1—H1E	109.8	C5—C6—H6B	109.5
H1D—C1—H1E	108.2	S1—C6—H6B	109.5
O1—C2—N2	124.15 (10)	H6A—C6—H6B	108.1
O1—C2—C1	120.47 (9)	S1—C7—H7A	109.5
N2—C2—C1	115.37 (9)	S1—C7—H7B	109.5
N2—C3—C4	110.59 (8)	H7A—C7—H7B	109.5
N2—C3—C5	109.33 (9)	S1—C7—H7C	109.5
C4—C3—C5	110.53 (8)	H7A—C7—H7C	109.5
N2—C3—H3	108.8	H7B—C7—H7C	109.5
			
C3—N2—C2—O1	−15.40 (16)	C5—C3—C4—O3	93.47 (11)
C3—N2—C2—C1	163.44 (9)	N2—C3—C4—O2	153.99 (9)
N1—C1—C2—O1	−8.46 (15)	C5—C3—C4—O2	−84.80 (11)
N1—C1—C2—N2	172.66 (9)	N2—C3—C5—C6	−176.94 (9)
C2—N2—C3—C4	−62.19 (12)	C4—C3—C5—C6	61.10 (12)
C2—N2—C3—C5	175.89 (9)	C3—C5—C6—S1	177.94 (8)
N2—C3—C4—O3	−27.75 (13)	C7—S1—C6—C5	171.26 (9)

### L-Glycyl-L-methionine (Gly-Met) Hydrogen-bond geometry (Å, º)

**Table:**

*D*—H···*A*	*D*—H	H···*A*	*D*···*A*	*D*—H···*A*
N1—H11*N*···O3^i^	0.83 (2)	2.15 (2)	2.8513 (13)	143.0 (18)
N1—H11*N*···O1	0.83 (2)	2.15 (2)	2.6619 (13)	120.1 (16)
N1—H12*N*···O2^ii^	0.886 (19)	1.850 (19)	2.7275 (12)	170.7 (18)
N1—H13*N*···O3^iii^	0.849 (19)	1.86 (2)	2.7006 (13)	168 (2)
N2—H2*N*···O2^iv^	0.832 (19)	2.000 (19)	2.8225 (12)	170.0 (18)

Symmetry codes: (i) *x*+1, *y*, *z*; (ii) *x*+1, *y*+1, *z*; (iii) −*x*+1, *y*+1/2, −*z*; (iv) *x*, *y*+1, *z*.

### L-Methionyl-L-glycine (Met-Gly) Crystal data

**Table:**

C_7_H_14_N_2_O_3_S	*D*_x_ = 1.382 Mg m^−^^3^
*M**_r_* = 206.26	Mo *K*α radiation, λ = 0.71073 Å
Orthorhombic, *P*2_1_2_1_2_1_	Cell parameters from 9827 reflections
*a* = 5.2521 (3) Å	θ = 3.0–40.2°
*b* = 11.4126 (7) Å	µ = 0.31 mm^−^^1^
*c* = 16.5403 (10) Å	*T* = 90 K
*V* = 991.43 (10) Å^3^	Needle fragment, colourless
*Z* = 4	0.25 × 0.24 × 0.17 mm
*F*(000) = 440	

### L-Methionyl-L-glycine (Met-Gly) Data collection

**Table:**

Bruker Kappa APEXII DUO CCD diffractometer	6231 independent reflections
Radiation source: fine-focus sealed tube	5939 reflections with *I* > 2σ(*I*)
TRIUMPH curved graphite monochromator	*R*_int_ = 0.030
φ and ω scans	θ_max_ = 40.3°, θ_min_ = 2.2°
Absorption correction: multi-scan (SADABS; Krause *et al.*, 2015)	*h* = −8→9
*T*_min_ = 0.872, *T*_max_ = 0.950	*k* = −20→20
33593 measured reflections	*l* = −30→30

### L-Methionyl-L-glycine (Met-Gly) Refinement

**Table:**

Refinement on *F*^2^	Hydrogen site location: mixed
Least-squares matrix: full	H atoms treated by a mixture of independent and constrained refinement
*R*[*F*^2^ > 2σ(*F*^2^)] = 0.023	*w* = 1/[σ^2^(*F*_o_^2^) + (0.0356*P*)^2^ + 0.0451*P*] where *P* = (*F*_o_^2^ + 2*F*_c_^2^)/3
*wR*(*F*^2^) = 0.061	(Δ/σ)_max_ = 0.001
*S* = 1.06	Δρ_max_ = 0.44 e Å^−^^3^
6231 reflections	Δρ_min_ = −0.20 e Å^−^^3^
131 parameters	Absolute structure: Flack *x* determined using 2494 quotients [(*I*^+^)-(*I*^-^)]/[(*I*^+^)+(*I*^-^)] Parsons *et al.* (2013)
0 restraints	Absolute structure parameter: 0.011 (11)

### L-Methionyl-L-glycine (Met-Gly) Special details

**Table:**

Geometry. All esds (except the esd in the dihedral angle between two l.s. planes) are estimated using the full covariance matrix. The cell esds are taken into account individually in the estimation of esds in distances, angles and torsion angles; correlations between esds in cell parameters are only used when they are defined by crystal symmetry. An approximate (isotropic) treatment of cell esds is used for estimating esds involving l.s. planes.

### L-Methionyl-L-glycine (Met-Gly) Fractional atomic coordinates and isotropic or equivalent isotropic displacement parameters (Å^2^)

**Table:**

	*x*	*y*	*z*	*U*_iso_*/*U*_eq_	
S1	0.29353 (4)	0.65791 (2)	0.59184 (2)	0.01687 (4)	
O1	0.56607 (10)	0.47315 (6)	0.32954 (3)	0.01617 (10)	
O2	−0.07675 (10)	0.65590 (6)	0.16589 (4)	0.01625 (10)	
O3	0.24892 (10)	0.71309 (4)	0.08834 (3)	0.01128 (8)	
N1	0.29316 (11)	0.32887 (5)	0.42498 (3)	0.00925 (8)	
H11N	0.230 (3)	0.2667 (12)	0.4002 (8)	0.014*	
H12N	0.280 (3)	0.3155 (11)	0.4782 (8)	0.014*	
H13N	0.461 (3)	0.3287 (11)	0.4122 (7)	0.014*	
N2	0.21065 (12)	0.55612 (5)	0.27617 (3)	0.01213 (9)	
H2N	0.044 (3)	0.5631 (11)	0.2749 (8)	0.015*	
C1	0.37117 (14)	0.56727 (6)	0.50511 (4)	0.01293 (10)	
H1D	0.471236	0.613218	0.465519	0.016*	
H1E	0.475036	0.499402	0.522492	0.016*	
C2	0.12412 (13)	0.52445 (6)	0.46605 (4)	0.01108 (9)	
H2A	0.028903	0.593014	0.445308	0.013*	
H2B	0.017812	0.486494	0.507971	0.013*	
C3	0.16563 (12)	0.43738 (5)	0.39633 (3)	0.00966 (9)	
H3	−0.003367	0.416245	0.372606	0.012*	
C4	0.33345 (12)	0.48982 (6)	0.32997 (4)	0.01035 (9)	
C5	0.34421 (13)	0.61524 (6)	0.21120 (4)	0.01259 (10)	
H5A	0.459462	0.559260	0.183793	0.015*	
H5B	0.448980	0.679543	0.233724	0.015*	
C6	0.15608 (12)	0.66476 (6)	0.15042 (3)	0.00962 (9)	
C7	0.59992 (18)	0.66260 (8)	0.64043 (6)	0.02365 (16)	
H7A	0.662481	0.582565	0.648561	0.035*	
H7B	0.584132	0.701884	0.692882	0.035*	
H7C	0.719997	0.705915	0.606324	0.035*	

### L-Methionyl-L-glycine (Met-Gly) Atomic displacement parameters (Å^2^)

**Table:**

	*U*^11^	*U*^22^	*U*^33^	*U*^12^	*U*^13^	*U*^23^
S1	0.01555 (8)	0.01596 (7)	0.01911 (8)	0.00057 (6)	0.00040 (6)	−0.00632 (6)
O1	0.00866 (19)	0.0242 (2)	0.0157 (2)	0.00274 (18)	0.00070 (17)	0.00795 (19)
O2	0.00915 (19)	0.0230 (2)	0.0166 (2)	−0.00048 (18)	0.00041 (16)	0.01047 (19)
O3	0.0129 (2)	0.01408 (18)	0.00689 (15)	−0.00366 (14)	0.00063 (14)	0.00177 (14)
N1	0.00953 (18)	0.01015 (17)	0.00807 (17)	0.00097 (16)	−0.00063 (15)	0.00075 (14)
N2	0.0095 (2)	0.0172 (2)	0.00969 (19)	0.00194 (19)	0.00069 (17)	0.00605 (17)
C1	0.0123 (2)	0.0118 (2)	0.0147 (2)	−0.0001 (2)	0.0012 (2)	−0.00119 (19)
C2	0.0104 (2)	0.0112 (2)	0.0117 (2)	0.00167 (18)	0.00146 (18)	0.00075 (18)
C3	0.0085 (2)	0.0115 (2)	0.0090 (2)	0.00079 (17)	−0.00053 (16)	0.00217 (16)
C4	0.0096 (2)	0.0131 (2)	0.00842 (19)	0.00101 (18)	−0.00038 (17)	0.00254 (17)
C5	0.0094 (2)	0.0181 (3)	0.0103 (2)	0.0009 (2)	0.00068 (18)	0.00519 (19)
C6	0.0102 (2)	0.0109 (2)	0.00774 (19)	−0.00110 (18)	−0.00008 (16)	0.00155 (17)
C7	0.0228 (4)	0.0199 (3)	0.0283 (4)	−0.0025 (3)	−0.0075 (3)	−0.0061 (3)

### L-Methionyl-L-glycine (Met-Gly) Geometric parameters (Å, º)

**Table:**

S1—C7	1.7995 (9)	C1—H1D	0.9900
S1—C1	1.8150 (7)	C1—H1E	0.9900
O1—C4	1.2365 (8)	C2—C3	1.5379 (9)
O2—C6	1.2534 (9)	C2—H2A	0.9900
O3—C6	1.2635 (8)	C2—H2B	0.9900
N1—C3	1.4855 (8)	C3—C4	1.5297 (9)
N1—H11N	0.885 (14)	C3—H3	1.0000
N1—H12N	0.896 (13)	C5—C6	1.5186 (9)
N1—H13N	0.907 (14)	C5—H5A	0.9900
N2—C4	1.3342 (8)	C5—H5B	0.9900
N2—C5	1.4499 (9)	C7—H7A	0.9800
N2—H2N	0.879 (15)	C7—H7B	0.9800
C1—C2	1.5296 (10)	C7—H7C	0.9800
			
C7—S1—C1	99.74 (4)	N1—C3—C2	111.31 (5)
C3—N1—H11N	110.5 (9)	C4—C3—C2	111.53 (5)
C3—N1—H12N	114.9 (8)	N1—C3—H3	108.9
H11N—N1—H12N	106.8 (12)	C4—C3—H3	108.9
C3—N1—H13N	111.5 (8)	C2—C3—H3	108.9
H11N—N1—H13N	105.0 (12)	O1—C4—N2	124.13 (6)
H12N—N1—H13N	107.6 (12)	O1—C4—C3	120.88 (6)
C4—N2—C5	121.62 (6)	N2—C4—C3	114.95 (6)
C4—N2—H2N	123.3 (8)	N2—C5—C6	110.43 (6)
C5—N2—H2N	115.0 (8)	N2—C5—H5A	109.6
C2—C1—S1	108.98 (5)	C6—C5—H5A	109.6
C2—C1—H1D	109.9	N2—C5—H5B	109.6
S1—C1—H1D	109.9	C6—C5—H5B	109.6
C2—C1—H1E	109.9	H5A—C5—H5B	108.1
S1—C1—H1E	109.9	O2—C6—O3	125.28 (6)
H1D—C1—H1E	108.3	O2—C6—C5	118.01 (5)
C1—C2—C3	113.76 (6)	O3—C6—C5	116.70 (6)
C1—C2—H2A	108.8	S1—C7—H7A	109.5
C3—C2—H2A	108.8	S1—C7—H7B	109.5
C1—C2—H2B	108.8	H7A—C7—H7B	109.5
C3—C2—H2B	108.8	S1—C7—H7C	109.5
H2A—C2—H2B	107.7	H7A—C7—H7C	109.5
N1—C3—C4	107.17 (5)	H7B—C7—H7C	109.5
			
C7—S1—C1—C2	165.35 (5)	C2—C3—C4—O1	93.98 (8)
S1—C1—C2—C3	−174.71 (4)	N1—C3—C4—N2	154.05 (6)
C1—C2—C3—N1	62.23 (7)	C2—C3—C4—N2	−83.89 (7)
C1—C2—C3—C4	−57.41 (7)	C4—N2—C5—C6	169.26 (6)
C5—N2—C4—O1	0.09 (11)	N2—C5—C6—O2	4.52 (9)
C5—N2—C4—C3	177.89 (6)	N2—C5—C6—O3	−176.26 (6)
N1—C3—C4—O1	−28.08 (9)		

### L-Methionyl-L-glycine (Met-Gly) Hydrogen-bond geometry (Å, º)

**Table:**

*D*—H···*A*	*D*—H	H···*A*	*D*···*A*	*D*—H···*A*
N1—H11*N*···O2^i^	0.885 (14)	1.854 (14)	2.7290 (8)	169.5 (13)
N1—H11*N*···O3^i^	0.885 (14)	2.593 (15)	3.1465 (8)	121.4 (11)
N1—H12*N*···O3^ii^	0.896 (13)	1.857 (13)	2.7531 (7)	179.6 (14)
N1—H13*N*···O3^iii^	0.907 (14)	2.015 (14)	2.7529 (8)	137.5 (11)
N1—H13*N*···O1	0.907 (14)	2.211 (13)	2.6940 (8)	112.7 (10)
N2—H2*N*···O2	0.879 (15)	2.185 (13)	2.6273 (8)	110.6 (10)

Symmetry codes: (i) −*x*, *y*−1/2, −*z*+1/2; (ii) −*x*+1/2, −*y*+1, *z*+1/2; (iii) −*x*+1, *y*−1/2, −*z*+1/2.
